# Human depopulation reshapes antimicrobial resistance dynamics in wildlife through ecological and transmission processes

**DOI:** 10.1016/j.onehlt.2026.101586

**Published:** 2026-09-16

**Authors:** Shiori Ikushima, Keita Fukasawa, Akira Yoshioka, Kahoko Tochigi, Hisashi Komatsu, Masahiko Kabeya, Manabu Onuma, Masanori Tamaoki, Seiji Hayashi

**Affiliations:** a National Institute for Environmental Studies, Fukushima Regional Collaborative Research Center, 10-2, Fukasaku, Miharu, Tamura, Fukushima 963-7700, Japan; b National Institute for Environmental Studies, Biodiversity Division, 16-2, Onogawa, Tsukuba, Ibaraki 305-8506, Japan; c Fukushima Prefectural Center for Environmental Creation, 10-2, Fukasaku, Miharu, Tamura, Fukushima 963-7700, Japan; d Fukushima Prefectural Wildlife Symbiosis Centre, 67, Nagakubo, Tamai, Otama, Fukushima 969-1302, Japan

**Keywords:** AMR, Camera trap, Depopulation, ESBL, Density-dependent transmission, Quinolone, Wildlife

## Abstract

Antimicrobial resistance (AMR) in wildlife is often interpreted as a spillover consequence of anthropogenic contamination, but the effects of long-term human depopulation remain poorly understood. We investigated quinolone-resistant *Escherichia coli* (QRE) and third-generation cephalosporin-resistant *E. coli* (3CRE) in wildlife and environmental samples from the Difficult-to-Return zones (DRZ), where human activity has been restricted since the 2011 Fukushima Daiichi Nuclear Power Plant accident, and from areas outside the DRZ (OUTSIDE), where human activity is present. Samples were collected from wild boar (*n* = 326), raccoons (*n* = 177), masked palm civets (*n* = 51), other wildlife (*n* = 39), wallows (*n* = 5), rivers (*n* = 9), and ponds (*n* = 9). Generalized additive models (GAMs) evaluated QRE occurrence using camera-trap-derived wildlife density, land-use variables, and livestock indices; core genome single-nucleotide polymorphism (cgSNP) analysis assessed genomic relatedness and transmission. QRE detection in wild boar was significantly higher in the DRZ, and cgSNP analysis indicated extensive clonal sharing among DRZ wild boar. GAM analysis further showed that QRE carriage increased with local wild boar density, supporting density-associated clonal expansion. In raccoons and masked palm civets, QRE carriage was associated with agricultural land, suggesting an influence of human-modified landscapes. Additionally, *bla*_CTX-M-15_ was disseminated across host species via a conserved mobile genetic element, suggesting horizontal gene transfer alongside clonal spread. These findings show that long-term human depopulation can reshape wildlife AMR dynamics and highlight the importance of integrating wildlife population management into One Health-based AMR risk assessments for human reinhabitation.

## Highlights

• Examines antimicrobial resistance in wildlife under long-term human depopulation.
• Density-dependent clonal spread may drive quinolone-resistant *E. coli* in wild boar
• Habitat type influences quinolone-resistant *E. coli* prevalence in mesocarnivores.
• *bla*_CTX-M-15_ genes disseminate via transferable genetic modules.

## Graphic abstract

**Figure:**
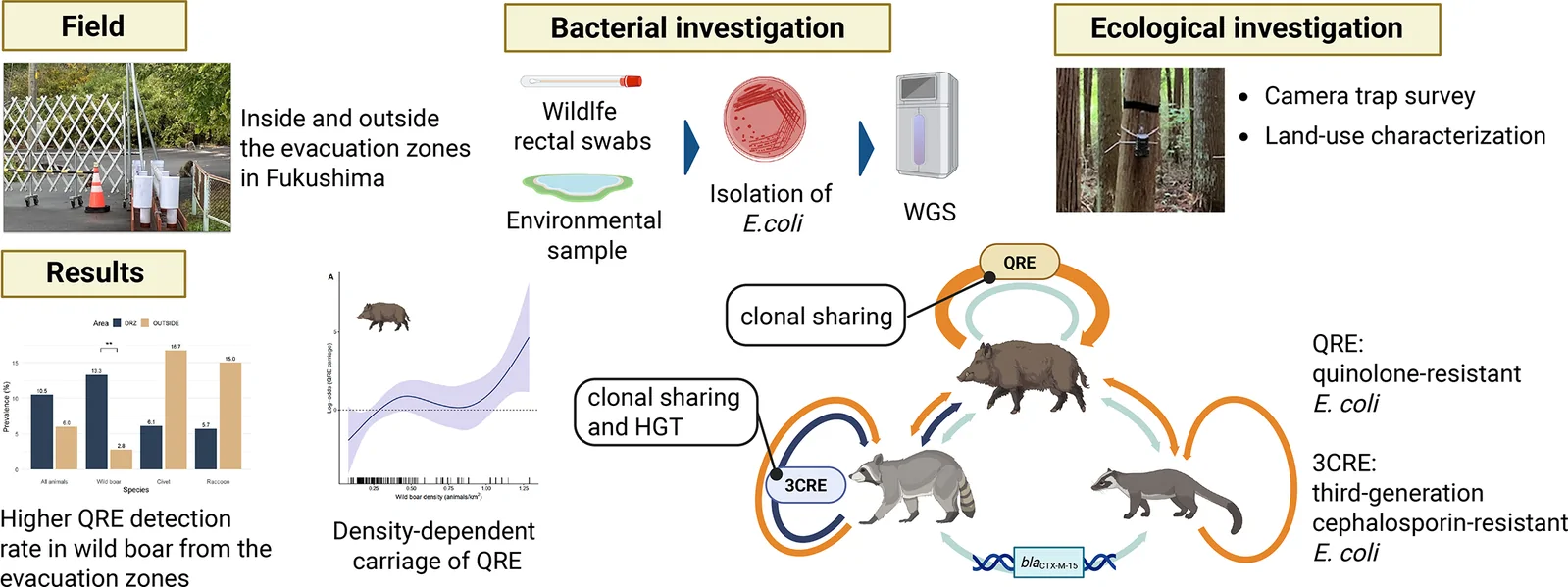

## 1 Introduction

Antimicrobial-resistant bacteria (ARB) pose a growing global threat to public health by increasing morbidity, mortality, and healthcare costs [1]. Because antimicrobial resistance (AMR) emerges and disseminates not only in human and veterinary clinical settings but also across agricultural and environmental domains, a One Health approach is needed to understand and address this issue [2].

Previous studies have suggested that the role of wildlife in the acquisition and dissemination of ARB varies across animal species and habitat types [3]. Accumulating evidence indicates that wildlife in proximity to human-inhabited areas is more likely to acquire clinically relevant ARB released from humans, particularly through exposure to medical wastewater, sewage, or manure [4]. However, there is a serious lack of knowledge regarding how human societal changes, such as rural depopulation or land-use shifts, affect the ecology of AMR in wildlife. Rural depopulation can have contrasting ecological consequences, including rewilding and land-use change [5], [6], [7], which may alter wildlife abundance, movement, and contact patterns. Yet, its public health implications remain unclear. To the best of our knowledge, no study has explicitly examined how long-term human depopulation influences AMR ecology in wildlife communities.

Following the Fukushima Daiichi Nuclear Power Plant (FDNPP) accident in 2011, large areas surrounding the plant were evacuated, resulting in long-term uninhabitation. The absence of humans and livestock has reshaped wildlife activity, with animals occupying abandoned landscapes and, in some cases, increasing in abundance [8]. This provides a quasi-experimental landscape for examining the ecological consequences of human absence, including potential effects on the distribution and transmission of ARB.

Quinolones and third-generation cephalosporins are classified by WHO as Highest Priority Critically Important Antimicrobials for human medicine and are used to treat bacterial infections in both humans and animals [9], although their use in animals is subject to prudent-use recommendations [10]. Quinolones inhibit bacterial DNA replication by targeting DNA gyrase and topoisomerase IV, and resistance in *Escherichia coli* (*E. coli*) is commonly associated with chromosomal mutations in the quinolone-resistance determining regions (QRDRs) [11]. In contrast, third-generation cephalosporins are β-lactam antimicrobials that inhibit bacterial cell-wall synthesis by targeting penicillin-binding proteins, whereas resistance in *E. coli* is commonly mediated by β-lactamases, including plasmid-borne ESBLs and AmpC-type enzymes [12]. Thus, comparison between quinolone-resistant *E. coli* (QRE) and third-generation cephalosporin-resistant *E. coli* (3CRE) may provide insight into AMR dynamics involving chromosomal mutation-mediated resistance and mobile genetic elements.

In Japan, fluoroquinolone resistance and ESBL production are clinically important resistance phenotypes in *E. coli*, particularly among isolates causing urinary tract infections [13]. In Fukushima, levofloxacin- and cefotaxime (CTX)-resistant *E. coli* have been detected among clinical isolates, with proportions slightly below the national averages (26.9% vs. 32.4% and 14.1% vs. 22.2%, respectively) [14]. Livestock production is extremely limited in municipalities that include the Difficult-to-Return zones (DRZ) in Fukushima, whereas cattle farming and poultry production are relatively common in areas outside the DRZ (OUTSIDE); in Japanese cattle and poultry, tetracyclines are the most commonly used antimicrobials, followed by penicillins and macrolides, with fluoroquinolones and cephalosporins also in use [15]. Thus, Fukushima provides a useful setting for examining wildlife- and environment-associated QRE and 3CRE in the context of clinical and livestock-associated AMR.

In this context, this study aimed to investigate the spatial distribution of two clinically relevant ARB, QRE and 3CRE, in wildlife inhabiting the DRZ and OUTSIDE in Fukushima Prefecture. We evaluated the ecological and anthropogenic drivers shaping the spatial distribution of QRE and 3CRE and further examined potential transmission among wildlife hosts.

## 2 Materials and methods

### 2.1 Study area and sample collection

The DRZ is located within approximately 30 km of the FDNPP (*37.420447°*N*, 141.033295°*E) and covers a total area of 309 km^2^. Following the 2011 accident, human residence and routine activities in the DRZ have been markedly restricted, resulting in long-term depopulation. In this study, OUTSIDE refers to areas of Fukushima Prefecture outside the DRZ where human activities, including residential, agricultural, and livestock-related activities, have been present. Culling of three generalist species, Japanese wild boar (*Sus scrofa leucomystax*), raccoon (*Procyon lotor*), and masked palm civet (*Paguma larvata*) has been performed by the Ministry of the Environment (MOE) to reduce overabundant populations in the DRZ. The estimated annual culling rate of wild boars in the DRZ was 19% in 2021 [16]; comparable estimates were unavailable for other species. At OUTSIDE, the animals were captured under municipal wildlife nuisance control programs. Culling activities for wildlife management were conducted throughout the year across the study region. No animals were killed for the purpose of the study.

Rectal swab samples were collected from 408 animals from June 2023 to July 2024 in the DRZ—including wild boar (*n* = 218), raccoon (*n* = 157), and civet (*n* = 33)—and from 185 animals from June 2023 to February 2025 in the OUTSIDE (across Fukushima Prefecture)—wild boar (*n* = 108), raccoon (*n* = 20), raccoon dog (*Nyctereutes procyonoides*, *n* = 21), civet (*n* = 18), crow (*Corvus* sp., *n* = 6), Japanese black bear (*Ursus thibetanus japonicus*, *n* = 4), sika deer (*Cervus nippon*, *n* = 3), Japanese badger (*Meles anakuma*, *n* = 3), and Japanese serow (*Capricornis crispus*, *n* = 2) (Fig. S1). Rectal swabs were collected using sterile swabs with Cary-Blair transport medium (Seed Swab γ1, Eiken Chemical, Tokyo, Japan), transported under cooled conditions, stored at 4 °C, and processed within one week. Cary-Blair medium has been reported to support recovery of antimicrobial-resistant *E. coli* and other enteric bacteria during refrigerated storage for approximately one week [17], [18], [19]. Rectal swabs were primarily collected from captured animals immediately after death. For two Japanese serows rescued and admitted to the Fukushima Prefectural Wildlife Symbiosis Center for rehabilitation, rectal swabs were collected non-lethally immediately after admission using the same sampling procedure as described above.

A total of 23 environmental samples were collected from wallows (five sites, two of which were soil samples), rivers (nine sites), and ponds (nine sites) within and adjacent to the DRZ between May and June 2025. Samples were classified according to the official DRZ boundary; three of the nine river sites were adjacent to, but outside, the DRZ and were, therefore, classified as OUTSIDE. Signs of animal activity, such as footprints and feces, were observed at 13 sites (wallows, 5/5; rivers, 2/9; and ponds, 6/9). The samples were transported to the laboratory under refrigerated ice and subsequently filtered through 0.45 μm membrane filters for bacterial isolation. For soil samples, 5.0 g of soil was suspended in 50 mL of sterilized distilled water in a sterile container and vigorously mixed. Subsequently, 100 μL of suspension was added to 10 mL of sterile distilled water and then filtered through a 0.45 μm membrane filter for bacterial isolation.

### 2.2 Bacterial isolation and antimicrobial susceptibility testing

Rectal swabs were inoculated onto desoxycholate‑hydrogen sulfide-lactose agar plates containing nalidixic acid (NAL) (50 μg/mL) or CTX (1 μg/mL) and a maximum of three colonies per animal were selected. Isolates were subjected to *E. coli* identification by PCR [20], with amplification performed under the following conditions: initial denaturation at 94 °C for 15 s; 35 cycles of 94 °C for 3 s, 50 °C for 10 s, and 74 °C for 35 s; followed by a final extension at 74 °C for 120 s. Antimicrobial susceptibility testing (AST) was performed by measuring the minimum inhibitory concentration (MIC) of ampicillin (1–128 μg/mL), cefazolin (1–128 μg/mL), CTX (0.5–64 μg/mL), meropenem (0.25–32 μg/mL), kanamycin (1–128 μg/mL), gentamicin (0.5–64 μg/mL), tetracycline (0.5–64 μg/mL), NAL (1–128 μg/mL), ciprofloxacin (CIP) (0.03–4 μg/mL), colistin (0.12–16 μg/mL), chloramphenicol (1–128 μg/mL), and sulfamethoxazol/trimethoprim (2.38/0.12–152/8 μg/mL) using a custom-made plate containing 100 μL Mueller Hinton broth in each well (Eiken Chemical, Tokyo, Japan). Approximately 2.5 μL of bacterial suspension was inoculated into each well, resulting in a final inoculum of approximately 5 × 10^4^ CFU/well. Plates were incubated at 35 °C for 16–20 h under static conditions. Resistance breakpoints were defined according to the guidelines of the Clinical and Laboratory Standards Institute [21]. Isolates resistant to quinolones (NAL and CIP) or third-generation cephalosporin (CTX) were examined for whole-genome sequencing (WGS). In principle, only one isolate was selected from each animal. However, when multiple isolates resistant to the same antibiotic group (up to three isolates per animal) were recovered from a single individual and exhibited distinct AST profiles, all isolates were subjected to WGS analysis.

### 2.3 Whole-genome sequencing and in silico analysis

For the 67 isolates (Table S1), whole-cell bacterial DNA was extracted using a phenol–chloroform method combined with bead beating and purified using the QIAquick PCR Purification Kit (QIAGEN, Hilden, Germany). DNA libraries were prepared using Illumina DNA Prep (Illumina, CA, USA) and quantified using the Qubit dsDNA HS Assay Kit (Thermo Fisher Scientific, MA, USA). Libraries were further quality-checked by the sequencing provider using qPCR-based quantification and fragment-size assessment with a Fragment Analyzer, and libraries passing QC were sequenced on the Illumina NovaSeq X Plus platform with paired-end 150-bp reads. Raw reads were quality-checked and trimmed using CLC Genomics Workbench v. 25.0.3 (Qiagen, Hilden, Germany) with the following parameters: quality trimming (limit = 0.01), removal of reads containing ambiguous nucleotides (max *N* = 0), automatic adapter trimming, and filtering of reads shorter than 30 bp. De novo assembly was then performed using the default parameters of the CLC de novo assembly tool. Assembly completeness was assessed using BUSCO v. 5.8.2 [22], and draft genomes were retained for further analysis if they met the following criteria: genome size of 4.5–5.5 Mbp, GC content of 50–51%, N50 > 50,000 bp, and complete BUSCO score > 99%.

Contig data were analyzed for acquired antimicrobial resistance genes (ARGs), virulence genes (VGs), and sequence types (STs) using ResFinder 4.7.2, VirulenceFinder 2.0, and MLST 2.0. Isolates were classified as extraintestinal pathogenic *E. coli* (ExPEC) if they carried two or more of *papA/papC*, *sfa-focDE*, *afa-draBC*, *iutA*, and *kpsMII*, and as uropathogenic *E. coli* (UPEC) if they carried three or more of *chuA*, *fyuA*, *vat*, and *yfcV*, according to previously established criteria [23]. For diarrheagenic *E. coli* pathotype-associated markers, isolates carrying Shiga toxin genes, including *stx1*, *stx2*, and their variants, were considered positive for Shiga-toxin producing *E. coli* (STEC)-associated markers [24], whereas isolates carrying enterotoxin genes encoding heat-labile toxin (*elt*) and/or heat-stable toxins (*estA* or *estB*) were considered positive for enterotoxigenic *E. coli* (ETEC)-associated markers [25], [26].

SNPs were identified using Snippy v. 4.6.0 [27] by aligning all isolates against the complete genome of *E. coli* str. K-12 substr. MG1655 (U00096.1). A clean core genome alignment was generated using snippy-core after removal of gaps and low-confidence sites, and recombinant regions were detected and masked using Gubbins v. 3.4.3 [28]. A maximum-likelihood phylogenetic tree was inferred from the Gubbins-filtered variable-site alignment of 246,976 sites using IQ-TREE v3.0.1 [29] under the GTR + F + ASC + G4 model with 1000 ultrafast bootstrap and 1000 SH-aLRT replicates. Pairwise SNP distances were calculated using snp-dists v. 0.8.2 (https://github.com/tseemann/snp-dists), and clonal groups were defined using a threshold of <100 SNPs [30].

To compare the genetic context of *bla*_CTX-M-15_, the flanking regions of this gene were aligned across all positive isolates using Easyfig 2.2.5 [31]. Reference sequences with high sequence identity were identified by BLAST and included for comparison.

### 2.4 Comparison of QRE and 3CRE detection rate within the area

The detection rates of QRE and 3CRE were compared between areas (DRZ vs. OUTSIDE) for all animals combined as well as for three representative species (wild boar, raccoon, and civet) using Fisher's exact test. A sample was considered positive only when colonies recovered from antimicrobial-containing agar were identified as *E. coli* and confirmed to be resistant by MIC testing. Samples showing no growth on antimicrobial-containing agar were considered negative.

### 2.5 Data preparation for generalized additive models (GAMs) for factors associated with QRE distribution

To identify the factors influencing the distribution of QRE in wild boar, raccoon, and civet (the primary animal species in this study), we fitted generalized additive models (GAMs). The dataset used for the GAM analysis is provided in Table S2. 3CRE was not modeled because of the small number of positive cases.

#### 2.5.1 Camera trap survey

For consistency with the GAM analysis, camera trap data were restricted to wild boars, raccoons, and masked palm civets. Camera trap surveys were conducted between May and October 2023 at 48 sites in eastern Fukushima Prefecture, including 12 within the DRZ. Bushnell Core DS-4 K cameras were installed in video mode at approximately 1 m above ground level.

From recorded videos, species identity and number of individuals were determined, and a relative abundance index (RAI; number of individuals per unit survey time) was calculated. Mean stay time within the effective detection zone and activity proportion were also derived for each species.

To estimate the spatial distribution of animal population densities as covariates in the GAM analysis of QRE carriage, we adopted a two-step procedure: (1) spatial smoothing of RAI and (2) conversion of smoothed RAI to population density using the random encounter and staying time (REST) model. For RAI smoothing, we applied a spatial generalized linear mixed model that incorporates an approximate Gaussian process model based on a stochastic partial differential equation approach [32], [33], [34]. Because the REST model estimates density from encounter rates and staying time within the effective detection zone, individual identification and exclusion of repeated recordings of the same animal were not required. Complete details of camera trap survey, spatial smoothing of RAI, or density estimation are provided in Supplementary Methods S1.

#### 2.5.2 Spatial covariate preparation and extraction

The coordinates of capture points were recorded for each individual. As explanatory variables, we considered the density of each animal species, the intensity of agricultural and built-up land, and livestock houses. Agricultural and built-up land intensities were derived from the latest JAXA High-Resolution Land-Use and Land-Cover Map v23.12 (Fig. S2), whereas livestock-house density was calculated from the number of livestock houses per local administrative unit (Fig. S3). Agricultural and built-up land intensities and livestock houses were represented as Gaussian-smoothed surfaces using species-specific spatial scales based on previous studies on home-range size. The corresponding covariate values were extracted at each capture point to construct the dataset for modeling. Isolates from animals captured outside the raster coverage were excluded. Continuous variables were standardized to a mean of zero and a standard deviation of one. Complete details of the land-use data sources, livestock-house data processing, Gaussian smoothing parameters, spatial projection, raster processing, and covariate extraction are provided in Supplementary Methods S2.

### 2.6 GAM analysis of QRE carriage

We modeled the probability of QRE carriage using GAMs with a binomial distribution and logit link function. Separate models were constructed for wild boars (wild boar model) and for raccoons and civets combined (raccoon–civet model), with the latter pooled due to limited sample sizes and similar ecological traits as mesocarnivores, including comparable diets, habitat use, and home-range sizes. The explanatory variables included the densities of wild boars, raccoons, and civets; the intensities of agricultural and built-up land; and the intensity of livestock houses. The nonlinear effects of the explanatory variables were modeled using thin plate regression splines. Two-dimensional smooth latitudes and longitudes were included to account for residual spatial autocorrelation. The capture site was included as a random effect to account for repeated sampling. All the models were fitted using the mgcv package in R [35].

Model selection was based on the Akaike information criterion (AIC), using maximum-likelihood fits. The model with the lowest AIC was selected as the best model; when multiple models had ΔAIC <2, the most parsimonious model was selected. The best model was then refitted using restricted maximum likelihood. Model adequacy was assessed by checking concurvity and residual diagnostics using the DHARMa package [36].

### 2.7 Multivariate analysis of gene profiles

To evaluate whether the composition of ARG and VG differed by host animal species or sampling area category, non-metric multidimensional scaling (NMDS) plots were generated from the presence/absence profiles of ARG and VG based on the Jaccard distance using the vegan package [37]. Permutational multivariate analysis of variance (PERMANOVA) was conducted to assess the statistical significance of animal species and sampling area on ARG and VG compositions, using the adonis2() function with a marginal test (by = “margin”) and 999 permutations. Their interaction was additionally tested by including an animal species × sampling area interaction term in the PERMANOVA model. The homogeneity of dispersion among groups was assessed using the betadisper() function with 999 permutations for animal species, sampling area, and their combined groups. For visualization using envfit() function, genes with near-zero variance (i.e., present in only one isolate or in ≥60 isolates) were excluded to avoid vectors driven by rare or near-constant features. The remaining genes were fitted onto the NMDS ordination using envfit() with 999 permutations, and only vectors with Benjamini–Hochberg-adjusted *p* < 0.05 were plotted. Isolates from badger, raccoon dog, and crow were excluded from the analyses due to small sample size (*n* = 1, 1, and 2, respectively).

### 2.8 Association between radiocesium burden and QRE carriage

To assess whether individual radiation exposure is associated with the carriage of QRE, log-transformed muscle ^137^Cs concentrations were compared between animals with and without QRE in wild boars from the DRZ using the Wilcoxon rank-sum test. This analysis was conducted for individuals for which both QRE carriage status and ^137^Cs concentration data were available (174/218; QRE-positive, 24/29; QRE-negative, 150/189). Muscle ^137^Cs concentrations were obtained from our previous study [38], in which radiocesium levels were quantified using a high-purity germanium detector as described therein. These values were used as a proxy for individual radiation burden.

All statistical analyses were conducted using the R v. 4.5.0 [39].

## 3 Results

### 3.1 Detection rate of QRE and 3CRE among wildlife inhabiting the DRZ or the OUTSIDE

A total of 58 QRE, seven 3CRE, and two QRE-3CRE isolates were obtained from wildlife and environmental samples. The source distribution of these resistant isolates was as follows: QRE: wild boar (*n* = 35), raccoon (*n* = 13), civet (*n* = 5), river (*n* = 2), raccoon dog (*n* = 1), badger (*n* = 1), and crow (*n* = 1); 3CRE: wild boar (*n* = 3), raccoon (*n* = 3), and civet (*n* = 1); and QRE-3CRE: wild boar (*n* = 1) and crow (*n* = 1).

**Fig. 1:**
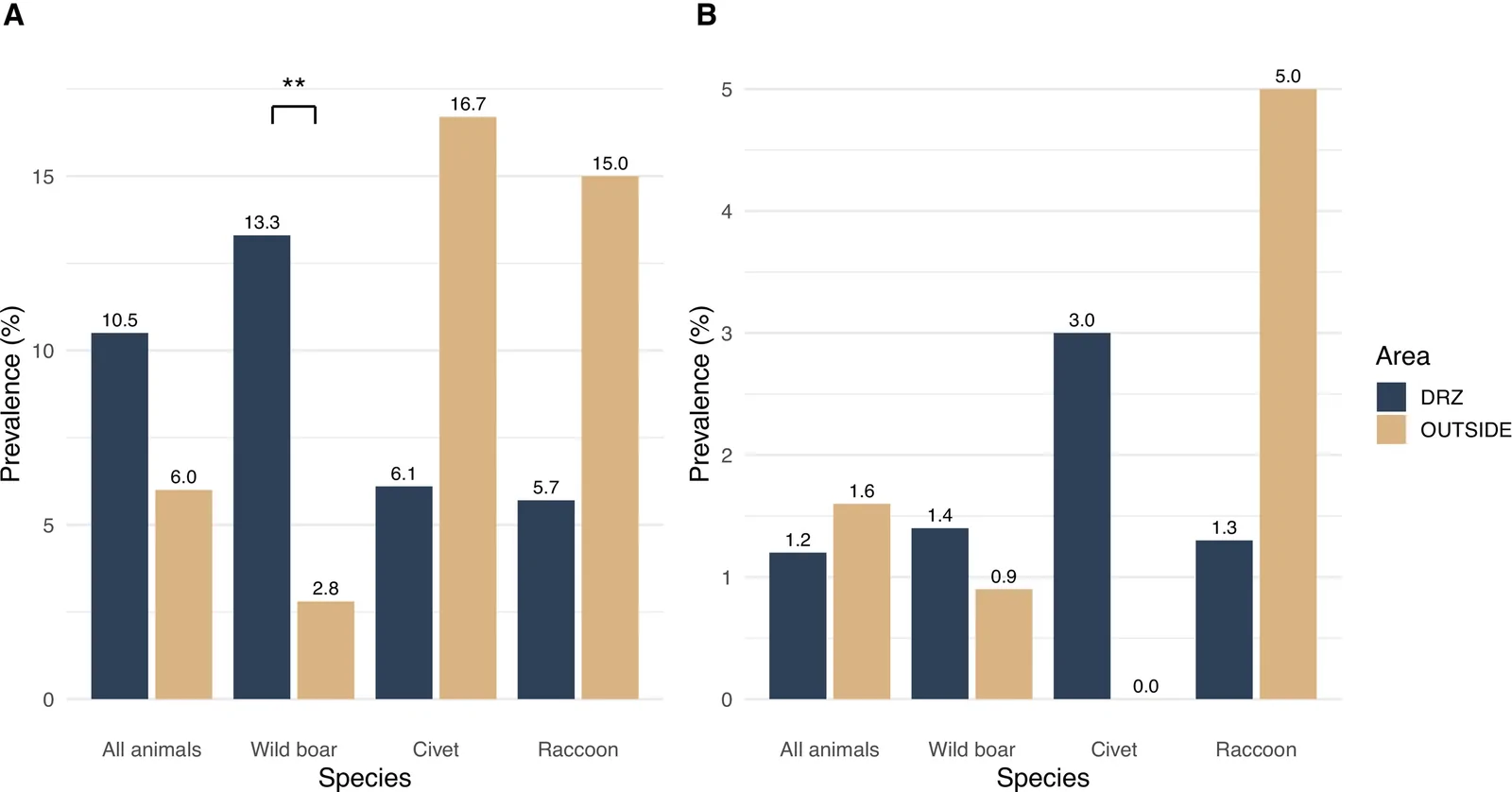
The detection rate of (A) quinolone-resistant and (B) third-generation cephalosporin-resistant *E. coli* in each wildlife species captured inside and outside the Difficult-to-Return zones (DRZ and OUTSIDE). Double asterisks** indicate statistical significance at *p* < 0.01 by Fisher's exact test.

Wild boars showed a significantly higher detection rate of QRE in the DRZ than in the OUTSIDE (13.3% vs. 2.8%, *p* < 0.01; Fig. 1A). In contrast, no significant differences were observed in QRE positivity in all animals combined (*p* = 0.090), raccoons (*p* = 0.14), or civets (*p* = 0.33). The detection rate of 3CRE was generally low (0–5%), and no significant differences were observed in any species (all animals, *p* = 1; wild boar, *p* = 1; raccoon, *p* = 0.30; civet, *p* = 1) (Fig. 1B).

### 3.2 The factors affecting the distribution of QRE among wildlife

**Fig. 2:**
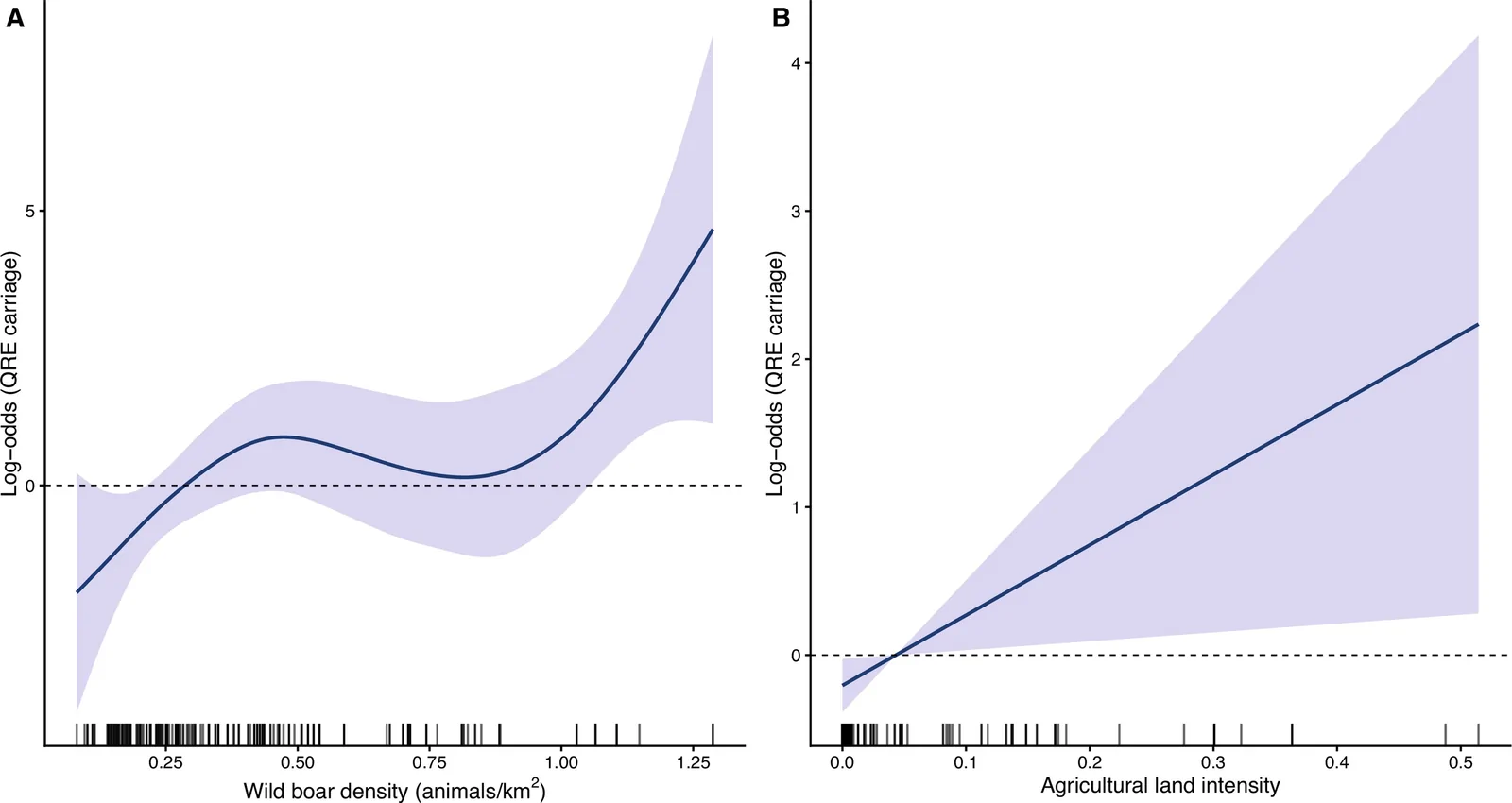
Partial effect of (A) wild boar density and (B) agricultural land intensity on the log-odds of quinolone-resistant *E. coli* carriage estimated using a generalized additive model (A, wild boar model; B, raccoon–civet model). The solid line represents the estimated smooth term, and the shaded area indicates the 95% confidence interval. The dashed horizontal line denotes zero effect on the log-odds scale. Rugs along the x-axis show the distribution of observed data.

For the wild boar model, the best-supported model included wild boar density and intensity of built-up land, with capture site included as a random effect (Table S3). Wild boar density showed a significant nonlinear association with the probability of QRE carriage (edf = 3.7, χ^2^ = 13.9, *p* = 0.012) (Table S4). The fitted smooth curve showed increasing trends in the probability of QRE carriage at wild boar densities below 0.5 animals/km^2^ and above approximately 0.8 animals/km^2^ (Fig. 2A).

For the raccoon–civet model, the best-supported model included intensities of agricultural land and built-up land, with capture site included as a random effect (Table S6). Agricultural land (edf = 1, χ^2^ = 5.0, *p* = 0.025) showed a significant positive association with the probability of QRE carriage (Fig. 2B; Table S7). For both models, diagnostic tests indicated no major violations of model assumptions (Table S5 and S8).

### 3.3 Core genome single-nucleotide polymorphism (cgSNP) analysis

**Fig. 3:**
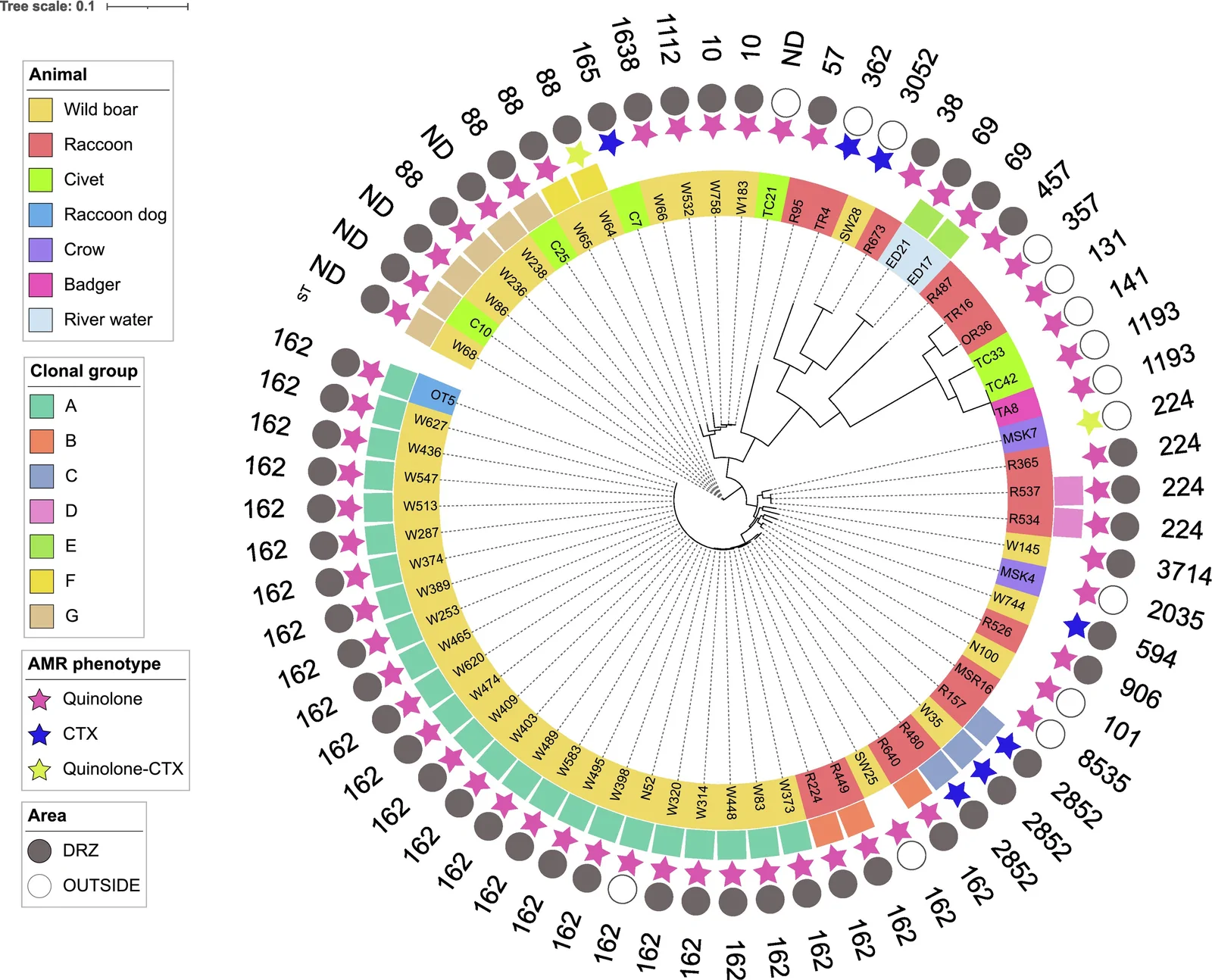
Core genome single-nucleotide polymorphism (cgSNP) based maximum likelihood phylogeny of quinolone-resistant or third-generation cephalosporin-resistant *E. coli* isolates. The phylogeny was inferred from a Gubbins-filtered cgSNP alignment of 246,976 variable sites using the GTR + F + ASC + G4 nucleotide substitution model. Isolates are colored by sample sources (Sample source) and annotated with clonal group defined by <100 cgSNPs (Clonal group), phenotypic AMR (AMR phenotype), sampling area (Area), and sequence type (ST). Scale bar indicates the number of substitutions per site.

Seven clonal groups (including both QRE and 3CRE) were identified, five of which had fewer than 20 SNPs (Fig. 3 and Fig. S4). Clonal group A (ST162) comprised 24 isolates, predominantly obtained from wild boars in the DRZ (*n* = 22), with single isolates from a raccoon dog in the DRZ and a wild boar in the OUTSIDE. Although the other clonal groups comprised fewer than six isolates, both QRE and 3CRE appeared to have been transmitted across animal species. The QRE isolates from environmental samples (river water) formed a distinct clonal group that was phylogenetically divergent from those isolated from wild animals.

### 3.4 Gene profile characteristics of QRE and 3CRE

Ten categories of AMR were identified (Fig. S5). Point mutations in QRDRs of *gyrA*, *parC*, and *parE* were the main mechanisms underlying quinolone resistance, although *qnr* family genes were also detected in some isolates. Clonal group A, which was the most widely distributed among individual hosts, harbored only a point mutation in *gyrA* and exhibited resistance only to nalidixic acid, whereas many other lineages were multidrug resistant.

**Fig. 4:**
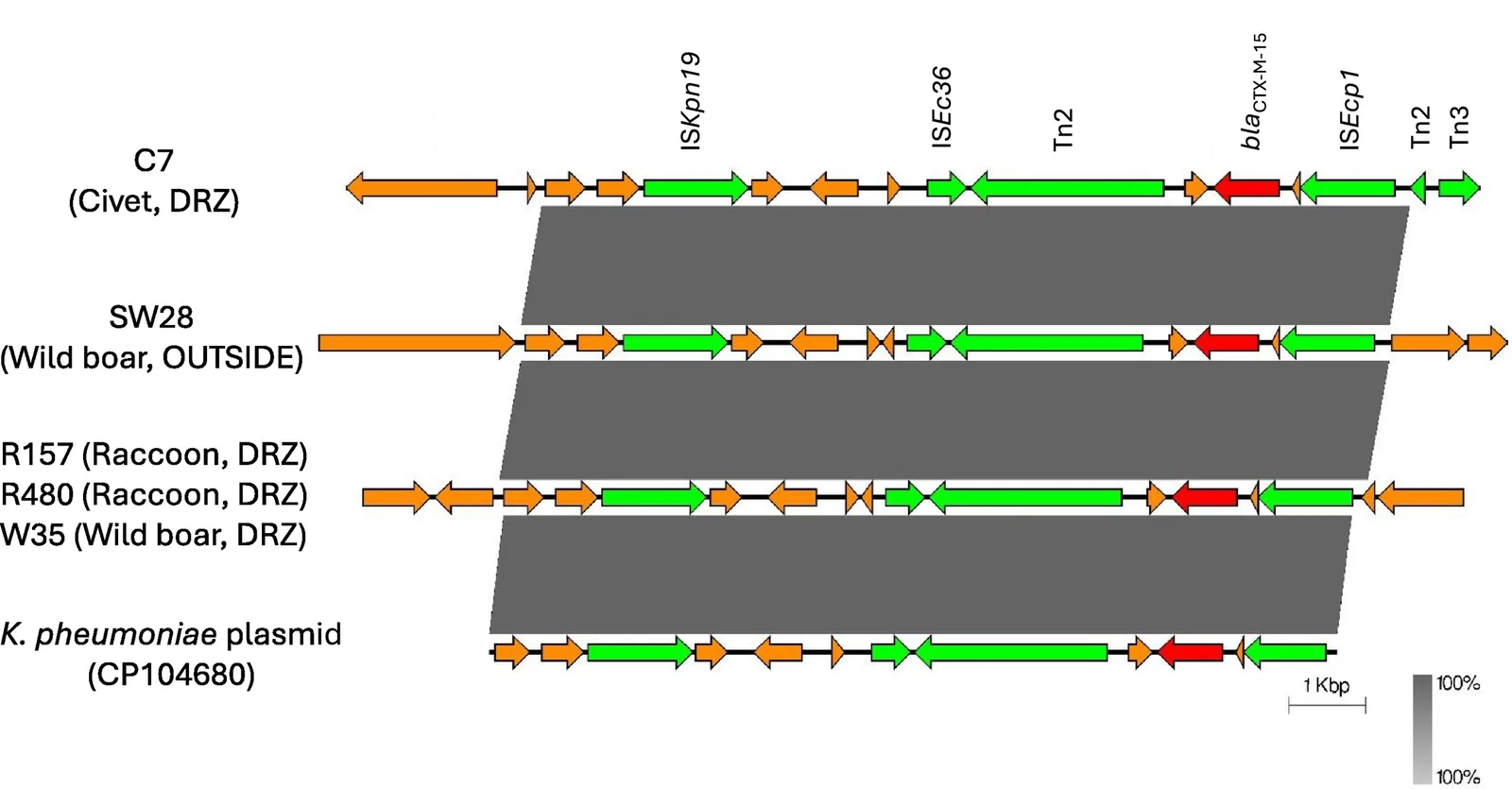
Flanking regions of the *bla*_CTX-M-15_ locus in third-generation cephalosporin-resistant *E. coli* isolates obtained in this study and a reference *Klebsiella pneumoniae* plasmid (accession no. CP104680). Genes are shown as arrows indicating transcriptional orientation; the antimicrobial-resistant gene is highlighted in red, the insertion sequence or transposon in green, and other predicted coding sequences, including hypothetical proteins, in orange. Gray shading indicates regions of nucleotide identity between sequences. The scale bar represents 1 kbp. (For interpretation of the references to colour in this figure legend, the reader is referred to the web version of this article.)

Regarding CTX-resistant determinants, *bla*_CTX-M-15_, *bla*_CTX-M-55_, *bla*_CTX-M-65_, *bla*_SHV-12_, and an *ampC* promoter mutation (-42C > T) were identified. Among these, *bla*_CTX-M-15_ was the only determinant detected in multiple isolates from different host species and sampling areas (Table S9). All *bla*_CTX-M-15_-positive isolates shared an identical genetic context consisting of an IS*Ecp1*–*bla*_CTX-M-15_–Tn2–IS*Ec36*–IS*Kpn19* composite module (Fig. 4). This module was also highly similar to that identified in a *Klebsiella pneumoniae* plasmid, suggesting dissemination of a conserved mobile genetic element across different genomic backgrounds.

Most isolates in clonal group A harbored ETEC-associated genes, predominantly *estB-STb1* (22/24) and, in one isolate, *eltIIAB-c2* (1/24), but lacked ExPEC-, UPEC-, or STEC-associated markers (Fig. S6). Several isolates from other clonal groups met the ExPEC criteria, whereas UPEC or STEC isolates did not belong to major clonal groups. ExPEC isolates belonged to ST88, ST162, ST1193, ST131, ST69, ST57, ST141, and ST906 (Table S1).

**Fig. 5:**
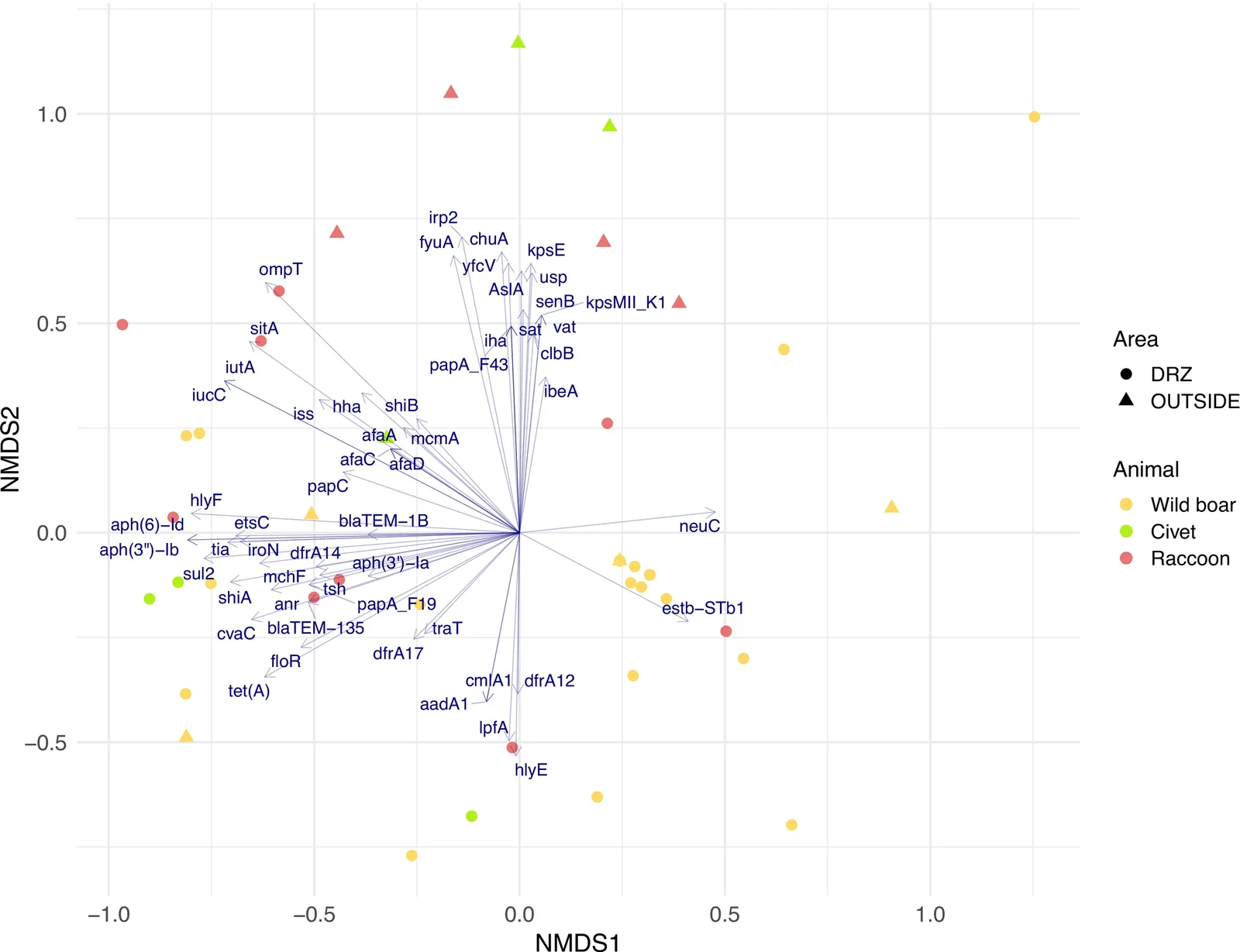
Non-metric multidimensional scaling (NMDS) based on Jaccard distances of antimicrobial resistance and virulence gene presence/absence profiles. Points represent isolates shaped by sampling area and colored by host species. Navy blue arrows indicate genes significantly associated with the ordination (envfit(), permutation test, *p* < 0.05). (For interpretation of the references to colour in this figure legend, the reader is referred to the web version of this article.)

PERMANOVA based on Jaccard dissimilarity showed that ARG and VG composition was significantly but weakly associated with host species and sampling area in the additive model, after mutual adjustment of the two variables (host species: R^2^ = 0.094, F = 3.19, *p* < 0.01; sampling area: R^2^ = 0.042, F = 2.87, *p* < 0.01). In addition, the host species × sampling area interaction was significant, although the effect size was modest (R^2^ = 0.057, F = 2.01, *p* = 0.018), suggesting that host-associated differences in ARG/VG composition were not entirely consistent across sampling areas. Tests for homogeneity of multivariate dispersion showed no significant differences among host species (F = 3.15, *p* = 0.054), sampling areas (F = 1.49, *p* = 0.21), or host species–sampling area groups (F = 1.12, *p* = 0.39). NMDS ordination did not reveal clearly separated clusters by host species or sampling area, consistent with the small PERMANOVA effect sizes (Fig. 5).

### 3.5 Association between muscle ^137^Cs concentration and QRE carriage in wild boar

There was no significant difference in log-transformed muscle ^137^Cs concentrations between QRE-positive and -negative wild boar from the DRZ (*p* = 0.79). The distributions of muscle ^137^Cs concentrations largely overlapped between QRE-positive and -negative animals (Fig. S7), despite a wide range of ^137^Cs concentrations observed.

## 4 Discussion

Previous meta-analyses have highlighted limitations in wildlife AMR studies, including insufficient characterization of habitat, host population density, and anthropogenic gradients, resulting in inconclusive assessments of human-associated drivers [3]. Here, by integrating host density, land use, and genomic epidemiology, we show that AMR dynamics in wildlife can be characterized by both ecological and anthropogenic factors. In contrast to previous studies suggesting that AMR in wildlife often reflects exposure to human-associated environments [4], [40], [41], [42], the detection rate of QRE in wild boar was higher in the DRZ than in the OUTSIDE. cgSNP analysis suggested that this pattern was driven largely by within-population transmission in the DRZ rather than repeated independent acquisition, with 28 of 33 QRE isolates (85%) showing clonal sharing. This interpretation was further supported by GAM, which showed a positive association between QRE carriage and wild boar density, supporting density-dependent transmission. Notably, the predominant clonal lineage showed limited ARG and VG profiles; dissemination among wildlife does not necessarily depend on high pathogenicity or multidrug resistance. Although epidemiological evidence of transmission between wildlife and environment was not obtained, the contribution of environmental reservoirs cannot be fully excluded, due to the limited number of environmental isolates analyzed and temporally offset from wildlife sampling.

The positive association between wild boar density and QRE carriage suggests that, even within the low-to-moderate density range observed in this study, increases in host density may enhance transmission opportunities, as expected under density-dependent transmission models [43]. This local transmission route is also compatible with reported movement patterns of wild boars in the DRZ, where monthly home ranges generally span several to approximately 15 km^2^ [16]. Thus, wild boar movements were most likely local and may not commonly connect the DRZ with livestock- or human wastewater-associated contamination sources in OUTSIDE. Following long-term human evacuation, wild boar populations in the DRZ have likely undergone ecological changes associated with rewilding, including increased abundance in the absence of human activity [8]. Although recent outbreaks of classical swine fever may have affected population size during the study period [44], the broader rewilding context likely contributed to the observed QRE transmission dynamics.

In raccoons and masked palm civets, QRE carriage was positively associated with agricultural land, suggesting that QRE exposure in these mesocarnivores may be linked to human-modified agricultural environments. Agricultural areas may function as ecological interfaces where wildlife encounters ARB through shared water systems, runoff, or other contaminated environmental substrates [2]. Although the explanatory power of the raccoon–civet model was modest, this association suggests that human-modified landscapes may contribute to QRE exposure in these species, while the underlying transmission routes remain unresolved.

The weak but significant associations with host species, sampling area, and their interaction may have been partly driven by localized clonal sharing, particularly among wild boar in the DRZ, which could make ARG/VG profiles more similar within specific host–area combinations. At the same time, the absence of clearly separated clusters suggests that ARG/VG profiles were also shaped by broader *E. coli* diversity and the presence of some shared ARGs across host categories, as reported in wild animal-derived *E. coli* [23]*.*

ExPEC isolates from wildlife and river water in both the DRZ and OUTSIDE included globally disseminated ExPEC lineages, such as ST131, ST88, ST69, and ST1193, which were ranked among the top 20 pandemic ExPEC lineages [45]. Consistent with the One Health perspective that ExPEC can occur across human, animal, and environmental reservoirs [46], this finding suggests that clinically relevant *E. coli* lineages may persist in wildlife and the environment even in depopulated areas, although the direction of transmission or temporal persistence could not be inferred from our data.

The conserved *bla*_CTX-M-15_-associated mobile element detected across isolates from different host species and sampling areas suggests that horizontal dissemination of a specific mobile resistance unit, rather than expansion of a single lineage alone, contributed to the distribution of CTX resistance in this ecosystem. The presence of *bla*_CTX-M-15_ together with IS*Ecp1*, which mediates the mobilization and expression of *bla*_CTX-M_ genes [47], suggests that this resistance unit can be transferable across diverse genomic backgrounds. The presence of a highly similar module in *Klebsiella pneumoniae* further supports its mobility beyond a single lineage or bacterial species. Its occurrence within and beyond the DRZ suggests that clinically relevant transferable resistance elements can circulate even under reduced direct anthropogenic pressure.

Although the potential effects of radiation on AMR dynamics in Fukushima wildlife cannot be entirely excluded, our results suggest that ecological factors played a more substantial role. QRE isolates in the DRZ were largely clonal, which is more consistent with persistence and transmission of existing lineages than with repeated de novo emergence. No association was detected between muscle ^137^Cs concentration and QRE carriage, and previous experimental studies have not supported an increase in AMR in both Gram-negative and Gram-positive bacteria, including *E. coli*, under radiation exposures relevant to Fukushima [48]. Together, these findings do not support radiation as a primary driver of the observed AMR patterns.

## 5 Conclusion

This study demonstrates that AMR in wildlife can be sustained by population-level ecological processes under long-term human depopulation, rather than being solely a legacy of anthropogenic contamination. In the DRZ, the observed QRE pattern in wild boar was consistent with density-dependent transmission, suggesting that wild boars can function as AMR reservoirs. These findings suggest that rewilding and altered population structure following human evacuation may create conditions conducive to ARB persistence. They also highlight the importance of accounting for host taxa and ecology in wildlife AMR monitoring and of integrating wildlife population management into the One Health framework in regions undergoing long-term depopulation and resettlement.

## Declaration of generative AI and AI-assisted technology in the manuscript preparation process

During the preparation of this work the authors used ChatGPT in order to improve the readability and language of the manuscript. After using AI tool, the authors reviewed and edited the content as needed and take full responsibility for the content of the published article.

## CRediT authorship contribution statement

**Shiori Ikushima:** Writing – review & editing, Writing – original draft, Visualization, Methodology, Investigation, Funding acquisition, Formal analysis, Data curation, Conceptualization. **Keita Fukasawa:** Writing – review & editing, Writing – original draft, Methodology, Investigation, Funding acquisition, Formal analysis, Data curation. **Akira Yoshioka:** Writing – review & editing, Funding acquisition. **Kahoko Tochigi:** Writing – review & editing, Formal analysis. **Hisashi Komatsu:** Writing – review & editing, Resources. **Masahiko Kabeya:** Resources. **Manabu Onuma:** Writing – review & editing, Funding acquisition. **Masanori Tamaoki:** Writing – review & editing, Funding acquisition. **Seiji Hayashi:** Writing – review & editing, Project administration.

## Funding resources

This work was supported by 10.13039/501100001691JSPS KAKENHI Grant Number 24K18034 and an internal competitive research fund of the 10.13039/501100005769National Institute for Environmental Studies. The funders had no role in the study design, data collection, analysis, interpretation, manuscript preparation, or decision to submit this article for publication.

## Declaration of competing interest

The authors declare that they have no known competing financial interests or personal relationships that could have appeared to influence the work reported in this paper.

## Data Availability

Data will be made available on request. Raw sequence reads of the *E. coli* genomes have been deposited in the DDBJ Sequence Read Archive (DRA) under accession numbers DRR900763–DRR900830.
