# Supplementary material for: Human depopulation reshapes antimicrobial resistance dynamics in wildlife through ecological and transmission processes
Source: One Health. 2026 Sep 16;23:101586. doi: 10.1016/j.onehlt.2026.101586 (PMC13602721; doi:10.1016/j.onehlt.2026.101586)
Supplement: Supplementary file 1 [file mmc1.docx]

**Supplementary Materials**

Supplementary Method S1: Camera trap survey and density estimation

Supplementary Method S2: Spatial covariate preparation and extraction

Figure S1-S7

Supplementary References

**Supplementary Method S1: Camera trap survey and density estimation**

**Camera trap survey**

For consistency with the GAM analysis, the camera-trap analysis was restricted to wild boars, raccoons, and masked palm civets, because these species accounted for the majority of samples collected within the DRZ and were widely distributed across Japan, making them suitable indicator species for AMR monitoring in wildlife. We conducted a camera trap survey to estimate animal density within and around the DRZ between May and October 2023. The 48 camera trap sites were established in national forests in eastern Fukushima Prefecture, of which 12 were within the evacuation zones. Of these, 36 sites were within 100 m of the locations of the previous camera trap monitoring sites reported by Fukasawa et al. [1]. To ensure that camera-trap locations were independent of animal trails; we randomly selected one of the six nearest tree trunks at each site. We installed a Bushnell Core DS-4K camera trap in video recording mode 1 m above the ground level.

For each recorded video, we identified the species, counted individuals, and calculated a media-based relative abundance index (RAI; number of individuals per unit of survey time). Furthermore, for wild boars, raccoons, and civets, we recorded whether the animals entered the effective detection zone (a regular triangular area of 1.57 m², following Nakashima et al. [2] and measured the time they stayed in the effective detection zone upon entry. One site outside the DRZ was excluded from further analysis due to a camera trap malfunction.

**Spatial smoothing of RAI**

To estimate the spatial distribution of animal population densities as covariates of AMR carriages, we adopted a two-step procedure: (1) spatial smoothing of media-based RAI and (2) conversion of RAI to population density using the REST model [3]. For RAI smoothing, we applied a spatial generalized linear mixed model (GLMM) that incorporates an approximate Gaussian process model based on a stochastic partial differential equation (SPDE) approach [4–6]. The SPDE-based approximation is a finite-element approximation of a Gaussian process with a Matérn covariance function. We assumed that the number of individuals photographed during the deployment period at each camera trap followed a Poisson distribution, with the log of its expected value expressed as the sum of the offset term for the log survey time and the SPDE Gaussian process. The smoothness parameter (ν) of the Matérn covariance function was set to 1, and the range parameter and conditional standard deviation were assigned penalized complexity priors [7]. The priors were set such that the probability of the range being less than 5 km was 0.01, and the probability of the conditional standard deviation exceeding three was 0.01. Parameter estimation was performed using the integrated nested Laplace approximation (INLA) implemented in the R package INLA. The triangular mesh for the finite element method was automatically generated using the inla.mesh.2d() function in the R package INLA, with edge lengths ranging from 1 to 5 km.

**Density estimation using the REST model**

The REST model [3] is an estimator of population density that uses video camera traps. Let *D_i_*, $\bar{y}_{i}$ and $\bar{T}_{i}$ denote the population density, mean number of encounters within the effective detection zone, and mean staying time in the detection zone at location i, respectively, which the REST estimator expresses as follows:
$D_{i}= \frac{\bar{y}_{i}\bar{T}_{i}}{s{rH}_{i}}$
where *s* is the area of the effective detection zone, *r* is the daily activity ratio of the species, and *H_i_* is the time the camera trap is in action. In this study, we introduce the following relationships and relevant parameters to transform the smoothed media-based RAI into the population density of the location. The mean number of encounters per survey time, $\bar{y}_{i}/H_{i}$, can be reasonably assumed to be proportional to the mean media-based RAI ($\bar{RAI}_{i}$) because whether an individual within the viewshed of a camera trap enters the effective detection zone or passes outside is random:
$\frac{\bar{y}_{i}}{H_{i}}=\alpha\bar{RAI}_{i}$.

where *α* is the rate of encounter to the effective detection zone per a media-based detection, which can be easily calculated by (Number of entries into the detection zone / Number of media-based detections). We then obtained the relationship between media-based RAI and animal density at site *i* as follows:

$D_{i}= \frac{{\alpha\bar{T}}_{i}}{sr}{\bar{\mathrm{RAI}}}_{i}$ (1)

The mean stay time and activity proportion were estimated for each species using camera trap data. The activity proportion was estimated using kernel density estimation with the fitact() function in the R package activity. While Nishimoto et al. [6] corrected for right-censored observations by assuming a parametric distribution for the mean staying time, we applied a design-based estimator [8] that used the observed mean staying time to avoid bias from incorrect distributional assumptions. The proportion of right-censored events was at most 0.0667 across the three species in this study, and censored observations had minimal impact on the mean staying time. Finally, the spatial distribution of animal density was obtained by combining the smoothed RAI from the Gaussian process model with Equation 1. The resulting densities were used as the local animal densities for each captured individual.

**Supplementary Method S2: Spatial covariate preparation and extraction**

The coordinates of capture points were recorded for each individual. As explanatory variables, we considered the density of each animal species, the intensity of agricultural and built-up land, and livestock houses. Agricultural and built-up land and livestock houses were represented as Gaussian-smoothed surfaces reflecting the proportion of each land-use type or the density of livestock houses. Given the extensive land-use modifications within the DRZ, including large-scale building removal, we used the latest version of the JAXA High-Resolution Land-Use and Land-Cover Map (v23.12) for calculating the intensity of agricultural and built-up land to ensure temporal consistency with the sampling period (10 m resolution; <https://www.eorc.jaxa.jp/ALOS/jp/dataset/lulc_j.htm>). Binary rasters for agricultural and built-up land were created using the JAXA map (presence = 1; absence = 0). We used the number of livestock houses in each local administrative unit (“Oaza”), standardized by the area of each Oaza. Referring to previous studies on home-range sizes of wild boars [9], raccoons [10,11], and civets [12,13], the intensity of agricultural and built-up land and livestock houses was calculated by applying Gaussian smoothing with σ = 1,000 m for wild boars and σ = 500 m for raccoons and civets. All spatial data were projected to the JGD2011 / UTM zone 54N (EPSG:6691), and raster layers were resampled to a 1,000 m grid resolution. The capture points of the animals were then overlaid on these rasters, and the corresponding values were extracted to construct the dataset for modeling. Isolates from animals captured in Koriyama and Minamiaizu were excluded because they fell outside the raster coverage. Continuous variables were standardized to a mean of zero and a standard deviation of one to improve model convergence and enable comparisons across variables with different scales.

**Figure S1**. Capture or sampling points of animals and environmental samples surveyed in this study (A, outline; B, zoom). The blue shaded area represents the Difficult-to-Return zones in Fukushima.

**Figure S2**. Distribution of major land-use categories in the study area. Land-use categories were extracted from the JAXA High-Resolution Land-Use and Land-Cover Map v23.12. Colored cells indicate 10 m resolution grid cells corresponding to each land-use category. Black points indicate sampling locations, and red outlines indicate the Difficult-to-Return zones (DRZ). Built-up land within the DRZ represents abandoned houses and infrastructure rather than currently inhabited residential areas.

**Figure S3**. Livestock-operation density for cattle, swine, broilers, and layers was calculated for each Oaza administrative unit, a submunicipal unit in Japan, as the number of livestock operations divided by area and is shown as operations per km². Black points indicate sampling locations, and red outlines indicate the Difficult-to-Return zones.

**Figure S4**. Pairwise core genome single-polymorphism distances within each clonal group. Boxes, horizontal lines within boxes, and dots indicate the interquartile range, the median, and pairwise comparisons between isolates, respectively.

**Figure S5**. Antimicrobial resistance gene and antimicrobial susceptibility testing profiles of quinolone- or third-generation cephalosporin-resistant *E. coli* recovered from wildlife inside and outside the Difficult-to-Return zones (DRZ) and from other environmental sources (ED17 and ED21) in the DRZ in Fukushima. Isolates were color-coded by clonal group when applicable. In isolates C10, C25, W238, W68, and W86, *bla*_TEM-32_, _-20_, and _-1C_ were detected with identical contig, position, and identity (%) as *bla*_TEM-135_, whereas in isolate W758, *bla*_TEM-220_, _-126,_ _-106,_ and _-1B_ were detected with the same contig, position, and identity (%) as *bla*_TEM-135_. For colistin, no “susceptible” category was defined in CLSI criteria [14]. AMP, ampicillin; CFZ, cefazolin; CTX, cefotaxime; MEPM, meropenem; GEN, gentamicin; KAN, kanamycin; TET, tetracycline; NAL, nalidixic acid; CIP, ciprofloxacin; CST, colistin; CHL, chloramphenicol; SXT, trimethoprim-sulfamethoxazole.

**Figure S6**. Virulence gene profile of quinolone- or third-generation cephalosporin-resistant *E. coli* recovered from wildlife inside and outside the Difficult-to-Return zones (DRZ) and from other environmental sources (ED17 and ED21) in the DRZ in Fukushima. Isolates were color-coded by clonal group and, when applicable, by pathotype based on the presence of pathotype-associated virulence genes. ETEC, ExPEC, STEC, and UPEC represent enterotoxigenic *E. coli*, extraintestinal pathogenic *E. coli*, Shiga toxin-producing *E. coli*, and uropathogenic *E. coli*, respectively.

**Figure S7.** Comparison of muscle ^137^Cs concentrations in wild boar from the DRZ between individuals positive and negative for quinolone-resistant *Escherichia coli*. Boxes, horizontal lines within boxes, and dots indicate the interquartile range, the median, and the ^137^Cs concentration of each individual, respectively.

**Supplementary References**

[1] K. Fukasawa, Y. Mishima, A. Yoshioka, N. Kumada, K. Totsu, T. Osawa, Mammal assemblages recorded by camera traps inside and outside the evacuation zone of the Fukushima Daiichi Nuclear Power Plant accident, Ecol. Res. 31 (2016) 493–493. https://doi.org/10.1007/s11284-016-1366-7.

[2] Y. Nakashima, S. Hongo, K. Mizuno, G. Yajima, Z.C.B. Dzefck, 2022. Double-observer approach with camera traps can correct imperfect detection and improve the accuracy of density estimation of unmarked animal populations. Sci. Rep. 12, 2011. https://doi.org/10.1038/s41598-022-05853-0.

[3] Y. Nakashima, K. Fukasawa, H. Samejima, Estimating animal density without individual recognition using information derivable exclusively from camera traps, J. Appl. Ecol. 55 (2018) 735–744. https://doi.org/10.1111/1365-2664.13059.

[4] E. Krainski, V. Gómez-Rubio, H. Bakka, A. Lenzi, D. Castro-Camilo, D. Simpson, F. Lindgren, H. Rue, Advanced spatial modeling with stochastic partial differential equations using R and INLA, Chapman and Hall/CRC, Boca Raton, FL, USA, 2019. https://doi.org/10.1201/9780429031892.

[5] F. Lindgren, H. Rue, J. Lindström, An explicit link between Gaussian fields and Gaussian Markov random fields: the stochastic partial differential equation approach, J. R. Stat. Soc. Ser. B Stat. Methodol. 73 (2011) 423–498. https://doi.org/10.1111/j.1467-9868.2011.00777.x.

[6] M. Nishimoto, T. Miyashita, K. Fukasawa, 2024. Spatiotemporal smoothing of water quality in a complex riverine system with physical barriers. Sci. Total Environ. 948, 174843. https://doi.org/10.1016/j.scitotenv.2024.174843.

[7] G. A. Fuglstad, D. Simpson, F. Lindgren, H. Rue, Constructing priors that penalize the complexity of Gaussian random fields, J. Am. Stat. Assoc. 114 (2019) 445–452. https://doi.org/10.1080/01621459.2017.1415907.

[8] L. Garland, E. Neilson, T. Avgar, E. Bayne, S. Boutin, Random encounter and staying time model testing with human volunteers, J. Wildl. Manage. 84 (2020) 1179–1184. https://doi.org/10.1002/jwmg.21879.

[9] Ministry of the Environment, Report on the survey of wildlife distribution and capture activities in the Difficult-to-Return zone and surrounding areas (in Japanese), Ministry of the Environment, Tokyo, 2021.

[10] T. Ikeda, M. Asano, Y. Matoba, G. Abe, Present status of invasive alien raccoon and its impact in Japan, Glob. Environ. Res. 8 (2004) 124–131. https://doi.org/10.57466/ger.8.2_125.

[11] O. Kurashima, N. Niwase, Spacing pattern of feral raccoons (*Procyon lotor*) in Eniwa, Hokkaido (in Japanese with English abstract), Mammal. Sci. 38 (1998) 9–22. https://doi.org/10.11238/mammalianscience.38.9.

[12] A. Toriyabe, M. Saito, A case study of estimating the home range of a nonindigenous species, the masked palm civet, in a rural landscape in Shonai region, Yamagata (in Japanese with English abstract), Nat. Environ. Sci. Res. 33 (2020) 15–20. https://doi.org/10.32280/nesr.33.0_15.

[13] Y. Zhou, C. Newman, F. Palomares, S. Zhang, Z. Xie, D.W. Macdonald, Spatial organization and activity patterns of the masked palm civet (*Paguma larvata*) in central-south China, J. Mammal. 95 (2014) 534–542. https://doi.org/10.1644/13-MAMM-A-185.

[14] Clinical and Laboratory Standards Institute, CLSI M100: performance standards for antimicrobial susceptibility testing, 33rd ed., Clinical and Laboratory Standards Institute, Wayne, PA, USA, 2023.
